# MicroRNA miR-491-5p Targeting both TP53 and Bcl-XL Induces Cell Apoptosis in SW1990 Pancreatic Cancer Cells through Mitochondria Mediated Pathway

**DOI:** 10.3390/molecules171214733

**Published:** 2012-12-11

**Authors:** Rong Guo, Yi Wang, Wei-Ye Shi, Bin Liu, Sheng-Qi Hou, Li Liu

**Affiliations:** Department of Microbiology, Institute of Basic Medical Sciences, Chinese Academy of Medical Sciences & Peking Union Medical College, Beijing 100005, China

**Keywords:** miR491-5p, Bcl-xL, TP53, STAT3, PI-3K/Akt

## Abstract

MicroRNA (miRNA) actively participates in a broad range of cellular processes such as proliferation, differentiation, cell survival and apoptosis. Deregulated expression of miRNA may affect cell growth and eventually lead to cancer. In this study, we found that hsa-miR491-5p (miR491-5p) displays a significantly high level of expression in normal human pancreas tissue *versus* pancreatic cancer cells. Targeted site prediction indicated that both Bcl-XL and TP53 contain miR-491-5p recognizing sites in their 3' UTRs. Overexpression of miR-491-5p in the pancreatic cancer cell line SW1990 effectively inhibited both endogenous Bcl-XL and TP53 gene expressions. Mutagenesis at the seed match region of both targeted genes further confirmed the specificity of miR491-5p recognition. Cell proliferation rate was inversely related to the increased doses of miR-491-5p. Flow cytometric analysis showed that the proportions of total apoptotic and early apoptotic cells were significantly induced as the dose of miR491-5p increased. Moreover, a mechanistic study indicated that miR-R491-5p-mediated cell apoptosis was associated with the activation of intrinsic mitochondria mediated pathways. miR491-5p also markedly inhibited mitogenic signaling pathways such as STAT3 and PI-3K/Akt, but not Ras/MAPK. Thus, our results demonstrated that miR491-5p could effectively target both Bcl-xL and TP53 and induce cell apoptosis independent of TP53.

## 1. Introduction

Increasing evidence has shown that miRNA may exert its proliferation or anti-proliferation effects during normal cell growth, while abnormal expression of miRNA may cause various diseases, including human cancer [1]. Apoptotic analysis has been frequently used to define the function of a particular miRNA. Currently, two main apoptotic pathways, namely the extrinsic or death receptor pathway and intrinsic or mitochondrial pathway have been well established, while a third apoptotic pathway is found to be associated with perforin/granzyme B release by cytotoxic T lymphocytes at the targeted cell [2,3]. The extrinsic pathway is activated by the death receptor after specific ligand interaction, which results in the formation of the death-inducing signaling complex (DISC). DISC serves as a platform to activate the initiator of caspase 8 [4,5]. The intrinsic pathway is a mitochondria-initiated process and requires the assembly of an apoptosome complex in response to some apoptotic stimulus [6]. Members of the BCL-2 protein family can be categorized into three subfamilies which may play opposite roles. They include the anti-apoptotic proteins such as BCL2 and BCL-XL, the pro-apoptotic proteins such as BAX and BAK and the BH3-only containing subfamily such as BAD and BID [7]. Anti-apoptotic proteins BCL2 and BCL-XL inhibit pro-apoptotic proteins BAX and BAK by protein-protein interaction. The competitive interaction between BH3-only protein and BCL2 and BCL-XL is able to activate the pro-apoptotic proteins BAX and BAK [8]. BAX and BAK activation promotes mitochondrial outer membrane permeabilization (MOMP) to release cytochrome c into the cytosol [3]. Then, cytochrome c contacts with apoptotic protease activating factor 1 (APAF1), results in the conformational changes [9] of APAF1 and promotes formation of APAF1 hexa- and heptamers [10]. The complex of cytochrome c and APAF1 oligomer then serve as a signaling platform (also termed apoptosome) for the recruitment and activation of the caspase 9 [10]. Although the three apoptotic pathways have different initiators, they all activate the common executioners, caspase 3 and caspase 7, to induce cell apoptosis. 

Pancreatic cancer is a type of aggressive disease that causes high mortality in the human population without early signs of symptoms [11,12,13]. Biomarker screening for pancreatic cancer is important for early diagnosis and prognosis of the disease and has been paid a great deal of attention in recent years [14]. Several large studies involving miRNA microarray analysis on RNAs isolated from formalin-fixed paraffin embedded pancreatic cancer specimens as well as surgically resected pancreatic cancer tissues and their respective control tissues have identified at least 40 miRNAs that are significantly upregulated or downregulated [15,16,17,18,19,20]. Some differentially expressed miRNAs such as miR-21, miR-155 and miR-196a-2 might be positively correlated with tumor growth and poor prognosis [16,20], but no study has reported the expression changes of miR491-5p during pancreatic cancer development. MicroRNA miR491-5p was an accidental hit when we were looking for the effect of host miRNA on viral life cycle. Expression analysis indicated that miR491-5p had remarkably high levels of expression among the total RNAs isolated from normal human pancreas. We then hypothesized that miR491-5p might be differentially expressed during pancreatic cancer development. In this study, we found that miR491-5p was downregulated in pancreatic cancer cells. Overexpression of miR491-5p in the pancreatic cancer cell line SW1990 dramatically reduced cell growth and markedly induced cell apoptosis through a mitochondria mediated intrinsic pathway. The current study may generate valuable information for future diagnosis and treatment of human pancreatic cancer. 

## 2. Results and Discussion

### 2.1. The Expression Profile of miR491-5p and Its Targeting Site Prediction

**Figure 1:**
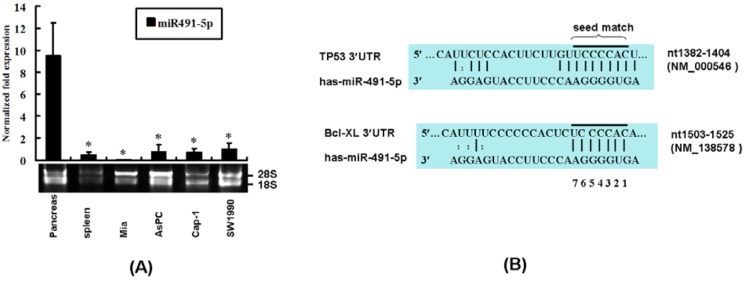
Expression analysis of miR491-5p and its predicted targeted sites. (**A**) Real time poly(A) qRT-PCR analysis on miR491-5p expressions in the total RNAs isolated from selected human tissues (spleen and pancreas) and four pancreatic cancer cell lines (Mia, AsPC, Cap-1 and SW1990). The integrity of RNA samples was assessed by gel electrophoresis to visualiz the presence of 18S and 28S RNAs. Data represented as mean ± SD from four independent experiments. The difference was calculated by student’s t test. Significant difference was indicated as * *p* < 0.01; (**B**) Targeted site prediction of miR491-5p on the 3' UTR of TP53 and Bcl-XL. The seed match region for each targeted gene was indicated.

Initial screening indicated that human normal pancreas expresses higher levels of miR491-5p. To study its function further, the endogenous levels of miR491-5p in the total RNAs freshly isolated from the selected human pancreatic cancer cell lines and tissues were assessed by real time poly(A) qRT-PCR. Interestingly, the expression level of miR491-5p in normal human pancreatic RNAs was significantly higher than that of the selected human pancreatic cancer cell lines Mia, AsPC-1, Cap-1 and SW1990 (Figure 1A), indicating that miR491-5p might play a role in pancreatic cancer development.

To search for miR491-5p targeting genes related to pancreatic cancer, three computer programs (DIANA-MICROT, MICRORNA and TARGETSCAN) were employed. We are looking for these targeted genes that were predicted to be positive with high scores by at least two different analysis tools. Finally, TP53 and Bcl-XL [21] were predicted to be the most likely targeted genes in pancreatic cancer by mR491-5p with appropriate seed matches (Figure 1B).

### 2.2. miR491-5p Effectively Downregulated TP53 and Bcl-XL Gene Expressions in Pancreatic Cancer Cells SW1990

**Figure 2:**
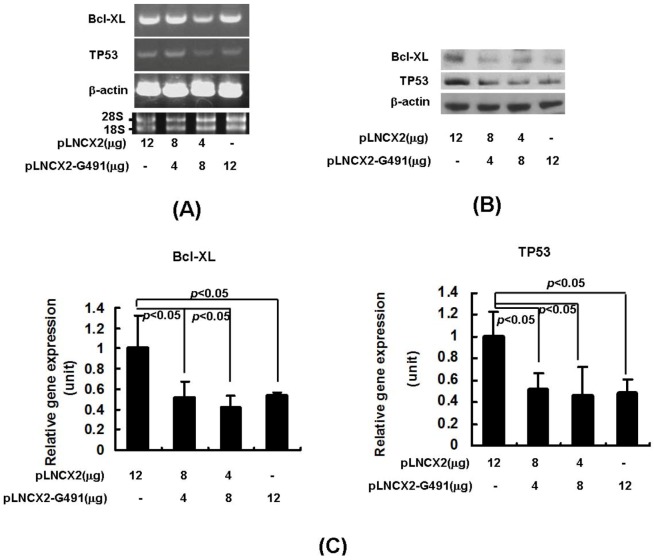
The down-regulation of the targeted gene expressions by plasmid derived miR491-5p in SW1990 cells. (**A**) Semi-quantitatative RT-PCR analysis on endogenous Bcl-XL and TP53 mRNA expressions was performed after transfecting SW1990 cells with increased doses of pLNCX2-G491. The integrity of RNA samples was assessed by gel electrophoresis to visualiz the presence of 18S and 28S RNAs; (**B**) Western blot analysis on pLNCX2-G491 transfected SW1990 cells. The reaction products were probed with anti-Bcl-XL and anti-TP53 antibodies. Beta-actin expression was served as loading control; (**C**) Real time qRT-PCR analysis on Bcl-XL and TP53 gene expressions was shown after delivering increased doses of pLNCX2-G491 into SW1990 cells. Beta-actin gene expression was served as internal control. Data represented as mean ± SD from four independent experiments. The difference was calculated by student’s t test. Significant difference was indicated as *p* < 0.05.

To test the effect of mR491-5p on its targeted gene expressions, the genome sequence containing pri-miR-491-5p (designed as G491) was first amplified from HEK293 genome DNAs and further inserted into the retroviral vector pLNCX2 to generate a pLNCX2-G491 expression construct. Overexpression of miR491-5p in SW1990 cells markedly decreased both endogenous TP53 and Bcl-XL mRNA levels as the delivered doses of pLNCX2-G491 increased (Figure 2A). Western blotting analysis further showed that the increased doses of pLNCX2-G491 effectively diminished the endogenous protein levels of both TP53 and Bcl-XL in SW1990 cells (Figure 2B). Real time quantitative RT-PCR (qRT-PCR) was employed to more accurately measure the mRNA levels of targeted gene expression. A dose dependent reduction in both TP53 and Bcl-XL mRNA expressions was observed as shown in Figure 2C.

**Figure 3:**
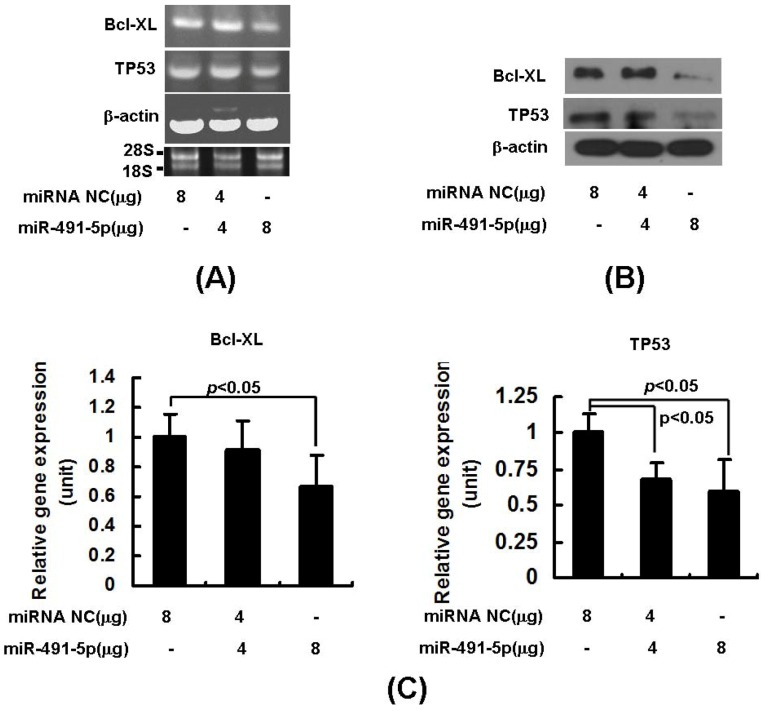
The down-regulation of the targeted gene expressions by chemically synthesized miR491-5p in SW1990 cells. (**A**) Semi-quantitatative RT-PCR analysis on endogenous Bcl-xL and TP53 mRNA expressions was performed after transfecting SW1990 cells with increased doses of miR491-5p. The integrity of RNA samples was assessed by gel electrophoresis to visualiz the presence of 18S and 28S ribosomal RNAs; (**B**) Western blot analysis on miR491-5p transfected SW1990 cells. The reaction products were probed with anti-Bcl-XL and anti-TP53 antibodies. Beta-actin expression was served as loading control; (**C**) Real time qRT-PCR analysis on Bcl-xL and TP53 gene expressions was shown after delivering increased doses of miR491-5p into SW1990 cells. Beta-actin gene expression was served as internal control. Data represented as mean ± SD from four independent experiments. The difference was calculated by student's t test. Significant difference was indicated as *p* < 0.05.

To further confirm the inhibitory effect induced by miR491-5p on targeted gene expression, we transiently transfected the chemically synthesized miR491-5p into SW1990 cells. Both mRNA and protein expressions of the endogenous TP53 and Bcl-XL gene were indeed significantly repressed as the higher doses of miR491-5p were delivered into the targeted cells (Figure 3A,B). Consistently, the results of real time qRT-PCR analysis quantitatively demonstrated that miR491-5p effectively down-regulate both TP53 and Bcl-XL mRNA levels in SW1990 cells (Figure 3C).

**Figure 4:**
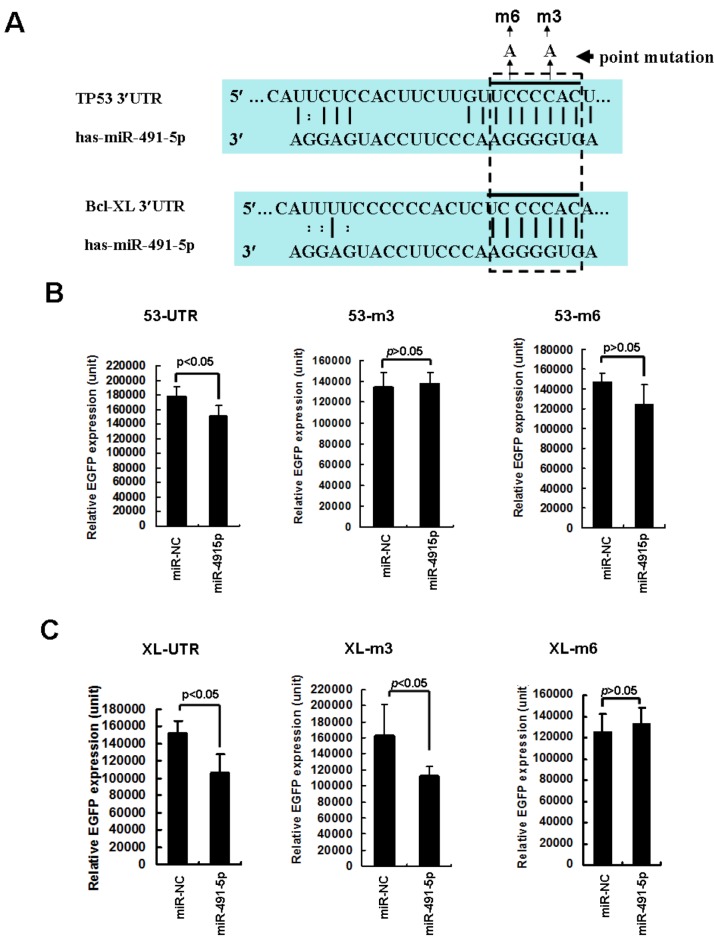
The rescued effects of point mutations at the seed regions of TP53 and Bcl-XL, the targeted genes by miR491-5p. (**A**) Two single C→A point mutations were generated at 3rd and 6th nucleotide of the seed region on the 3'UTRs of the targeted genes TP53 and Bcl-XL. These mutants were named as EGFP-53m3, EGFP-53m6, EGFP-XLm3 and EGFP-XLm6; (**B**) Flow cytometric analysis on the expression of EGFP gene cassette carrying wild type TP53 3'UTR (53-UTR) or its mutants (53-m3 and 53-m6). About 4 μg of synthesized miR491-5p was individually cotransfected with 1 μg of pEGFP-53-UTR or its mutants into SW1990 cells. Data represents as mean ± SD from three to four independent experiments. The difference was calculated by student’s t test. Significant difference was indicated as *p* < 0.05, while no significant difference was indicated as *p* > 0.05; (**C**) Flow cytometric analysis on the expression of EGFP gene cassette carrying the wild type Bcl-XL 3'UTR (XL-UTR) or its mutants (XL-m3 and XL-m6). About 4 μg of synthesized miR491-5p was individually cotransfected with 1 μg of pEGFP-XL-UTR or its mutants into SW1990 cells. Data represents as mean ± SD from three to four independent experiments. The difference was calculated by student’s t test. Significant difference was indicated as *p* < 0.05, while no significant difference was indicated as *p* > 0.05.

Moreover, the specificity of miR491-5p-mediated targeted gene repression was evaluated by creating single point mutation at 3rd (C→A) or 6th (C→A) nucleotide at the seed region (Figure 4A). The wild type and mutated recognition sites of miR491-5p on both TP53 and Bcl-XL 3'UTRs (53-UTR and XL-UTR) were RT-PCR isolated and inserted into the 3'UTRs of EGFP gene. The chemically synthesized miR491-5p significantly repressed both EGFP-53-UTR and EGFP-XL-UTR expressions (Figure 4B and Figure 4C). The 3rd nucleotide C→A mutation at the 53-UTR (simplified as 53-m3) as well as its 6th nucleotide C→A mutation (simplified as 53-m6) significantly rescued miR491-5p mediated targeted gene repression (Figure 4B), while mutant XL-m3 but not XL-m6 could effectively prevent miR491-5p mediated EGFP-XL-UTR gene repressions (Figure 4C). Overall, our data strongly indicates that both Bcl-XL and TP53 3'UTRs contain the real target sites that could be effectively recognized by miR491-5p.

### 2.3. Overexpression of miR491-5p Inhibited Cell Proliferation and Promoted Cell Apoptosis in SW1990 Cells

**Figure 5:**
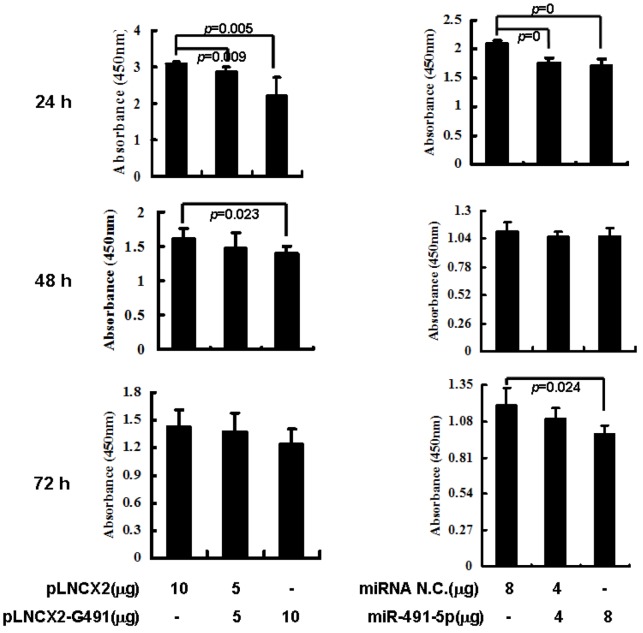
miR491-5p inhibited the proliferation of pancreatic cancer cell line SW1990. SW1990 cells were transfected with increased doses (0, 5 and 10 μg) of miR491-5p for 24, 48 and 72 h. At each time point, the transfected cells were collected and assayed by CCK-8 kit. The proliferation rates were measured at 450 nm with a plate reader. The values represented as the mean ± SD from six independent experiments for each dose at each assay point, that were subjected to student’s t test alaysis. The experiment included the empty vector (pLNCX2) transfected sample as a standard control. Difference was considerated significant if *p* < 0.05 or *p* < 0.01.

In principle, individually knocking down Bcl-XL and TP53 expressions in a given cell will generate antagonistic effects on cell growth. Since Bcl-XL is an anti-apoptotic gene, down-regulating Bcl-XL expression will activate the apoptotic pathway. In contrast, due to its tumor suppressor effect, inhibiting TP53 expression by miR491-5p in the pancreatic cancer cell will facilitate tumor growth. To detect which cellular process might play the predominant role in responding to exogenous miR491-5p, cell proliferation assay was performed after transiently transfected either pLNCX2-G491 or chemically synthesized miR491-5p into SW1990 cells. The results of CCK-8 assay demonstrated that at 24 h post-transfection, both pLNCX2-G491 and chemically synthesized miR491-5p significantly inhibited SW1990 cell growth in a dose dependent manner (Figure 5). However, after longer incubation, the significant inhibition on cell growth was only detected at the higher dose of pLNCX2-G491 (10 μg) and miR491-5p (8 μg) with an incubation time 48 h and 72 h, respectively. Thus, the results indicated that the apoptotic signal generated by miR491-5p mediated Bcl-XL repression might override that of the miR491-5p mediated downregulation of TP53 in SW1990 cells.

**Figure 6:**
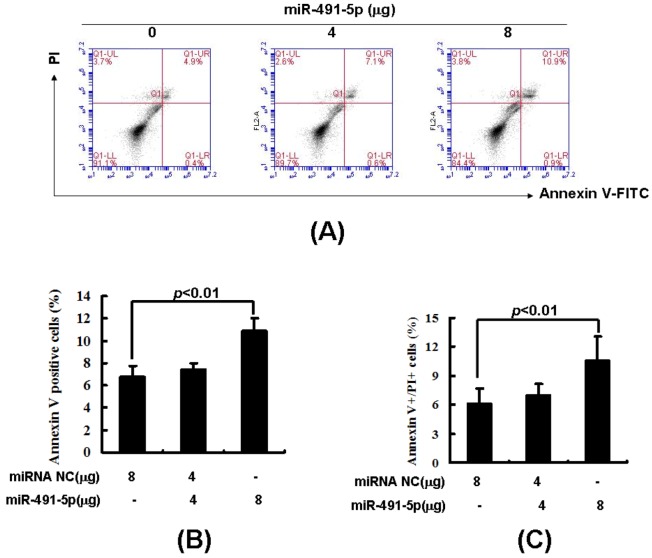
miR491-5p induced cell apoptosis in pancreastic cancer cell line SW1990. (**A**) The detection of cell apoptosis in miR491-5p transfected SW1990 cells was performed with Annexin V/PI double staining followed by flow cytometric analysis; (**B**) Quantitation of total apoptotic cells. After 48 h transfection, the transfected SW1990 cells were harvested and stained with Annexin V and PI. The reation product was subjected to flow cytometric analysis. Annexin V positive cells were counted. Each value represented as mean ± SD from four independent transfections. The empty vector (pLNCX2) transfected sample was served as a standard control. Student's t test was performed. Difference was considerated significant if *p* < 0.01; (**C**) Quantitation of late apoptotic cells. The transfected SW1990 cells were stained and analyzed as described in (**B**). Annexin V+/PI+ double positive cells were counted. Each value represented as mean ± SD from four independent transfections. The negative control miRNA transfected sample was served as a standard control. Student's t test was performed. Difference was considerated significant if *p* < 0.01.

To directly measure miR491-5p mediated apoptosis, Annexin V and PI double staining was performed to quantitate the cell proportions in early and late apoptosis. Flow cytometric analysis demonstrated an enhanced proportion of apoptotic cells as the delivered dose of miR491-5p increased (Figure 6A). Statistical analysis further showed that both the total and late apoptotic cells were significantly increased as the higher dose of miR491-5p was delivered (Figure 6B,C). Therefore, our data indicates that miR491-5p could induce a stronger apoptotic signal in SW1990 cells even at the TP53 gene under repression. 

### 2.4. miR491-5p Effectively Activated Intrinsic Mitochondrial Apoptotic Pathway

**Figure 7:**
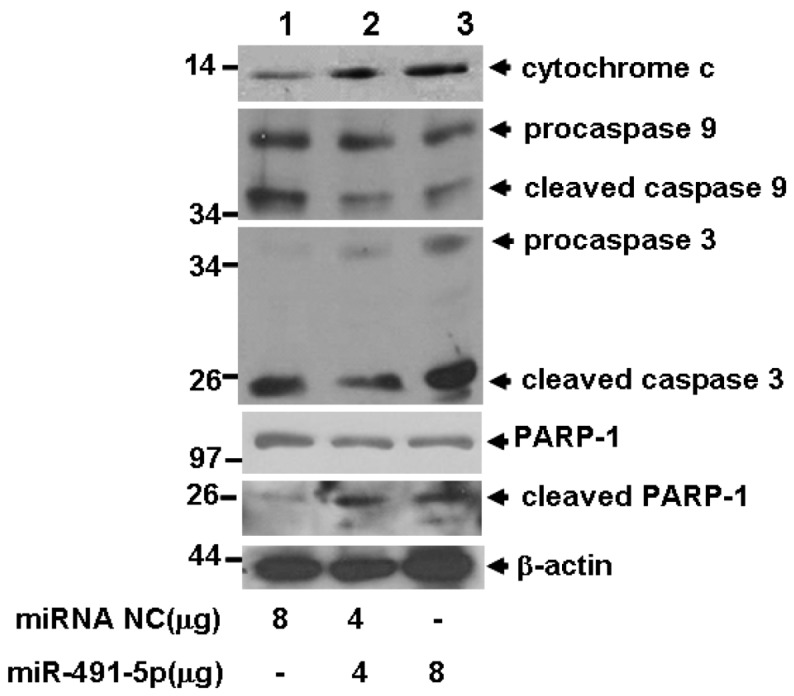
miR491-5p mediated cell apoptosis was associated with mitochrondia mediated intrinsic pathway. The cell lysates prepared from miR491-5p transfected SW1990 cells were probed with anti-cytochrome c, anti-caspase 3, anti caspase 9 and anti-PARP-1 antibodies and analyzed by Western blot analysis. Beta-actin gene expression was served as internal control.

Since Bcl-XL is an anti-apoptotic protein that plays key role in intrinsic mitochondrial pathway, downregulation of Bcl-XL expression by miR491-5p should activate the main components to this pathway. Indeed, increased delivery of miR491-5p into SW1990 cells enhanced the protein level of cytochrome c, indicating that the release of cytochrome c from mitochondrial was promoted. Also, increased delivery of miR491-5p subsequently activated the caspase cascade responsible for apoptotic initiation and execution by inducing the cleavage of procasepase 9, procasepase 3 and PARP-1 (Figure 7).

### 2.5. miR491-5p Inhibited both STAT3 and PI3K/Akt Signaling Pathways

**Figure 8:**
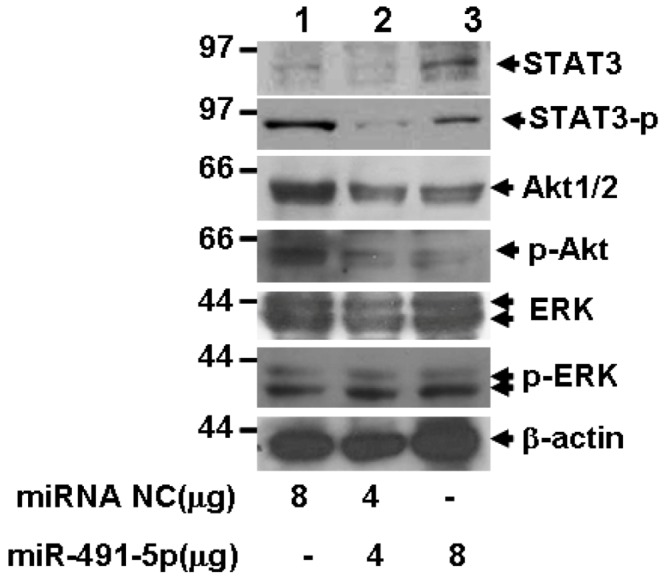
The potential signaling pathways affected by miR491-5p in SW1990 cells. Western blot analysis was performed using anti Akt, anti-phosphoryled Akt, anti ERK, anti-phosphorylated ERK, anti STAT3 and anti-phosphorylated STAT3 antibodies. Beta-actin gene expression was served as internal control.

Bcl-XL is an anti-apoptotic molecule that can be aberrantly overexpressed in some tumor cells [22,23,24,25] and is actively involved in multiple mitogenic signaling pathways [25,26]. Catlett-Falcone *et al.* demonstrated that Bcl-XL gene is the directly targeted gene of signal transducer and activator of transcription 3 (STAT3) [27]. Evidence is also shown that Bcl-XL might function cooperatively or synergistically with Akt to induce growth-factor independent growth and leukemogenic transformation or to contribute to the resistance of cell apoptosis during chemotherapy [28,29]. To test the effect of miR491-5p on the activation of the main mitogenic signaling pathways, western blot analysis was conducted to detect the phosphorylation levels of STAT3, Akt and ERK. Figure 8 shows that the activation of STAT3 and Akt was inhibited as the doses of miR491-5p increased. However, no significant alteration was observed in the phosphorylation level of ERK which was tested at the same condition, indicating that miR491-5p had a negative effect on both STAT3 and PI3K/Akt singling pathways.

## 3. Experimental

### 3.1. Cell Lines, Antibodies and Chemical Reagents

Human pancreatic adenocarcinoma cell lines SW1990, Mia PaCa-2 (Mia), Capan-1 (Cap-1), AsPC-1 were derived from the Cell Culture Center of Institute of Basic Medical Sciences, Chinese Academy of Medical Sciences (Beijing, China). Two human tissue RNAs (one from one normal pancreas and the other from the normal spleen of the same subject) were obtained from Cybrdi Biotechnology (Xi’an, China). Antibodies to β-actin, STAT3, phospholated STAT3, ERK1/2, phospholated ERK1/2, Akt, phospholated Akt, Caspase 3 were purchased from Santa Cruz Biotechnology (Santa Cruz, CA, USA). Antibodies to Caspase 9, TP53 and Bcl-XL were from Bioworld Technology, Inc (Minneapolis, MN). Antibody to Cytochrome C was purchased from Epitomcs, Inc (Burlingame, CA, USA). The peroxidase-conjugated secondary antibodies (goat anti mouse or rabbit IgG) were from Zhongshan Biotechnology (Beijing, China). Chemically synthesized has-miR491-5p and negative control were derived from Shanghai GenePharma., Ltd. (Shanghai, China).

### 3.2. The Construction of microRNA Expression Plasmid

To construct pLNCX2-G491, the pri-miR-491 sequence was first amplified from genomic DNA isolated from HEK293 cells with a pair of primers 5'-ttagatctacagaagctgcacacataca-3' and 5'-ttgtcgactatctcaactgctgccatca-3'. Then, the PCR products were subcloned into the T-A vector pMD-18T (Takara, Dalian, China) followed by sequencing confirmation. After restriction endonuclease digestion, the released 1483bp PCR product was subcloned into the BglII/SalI sites of pLNCX2 to form plasmid construct pLNCX2-G491.

### 3.3. Reverse Transcription-Polymerase Chain Reaction (RT-PCR) and Real Time Quantitative RT-PCR (qRT-PCR)

Total RNAs were extracted from the cultured cells with TRIzol (Invitrogen, Carlsbad, CA, USA). The concentrations of the purified RNAs were measured by NanoDrop^TM^ 2000 (Thermo Scientific, Pittsburgh, PA, USA), and the purity of the isolated RNAs were assessed by the ratio of OD260/OD280. Only RNA sample with OD260/OD280 ratio between 1.8~2.0 was used in the following RT-PCR analysis. The primers used in the RT-PCR reactions were listed in Table 1. One μg of total RNAs was subjected to RT-PCR analysis using PrimeScript One Step RT-PCR Kit (Takara Biotechnology, Dalian, China). One-step quantitative real time PCR (qRT-PCR) by SYBR Green approach (kit from Takara Biotechnology, Dalian, China) was also performed to monitor the targeted gene (TP53 and Bcl-XL) expressions. The level of β-actin gene expression was served as internal control. The relative quantifications on TP53 and Bcl-XL expressions were normalized by using the expression level of β-actin as a reference.The expression of miR491-5p was detected by poly(A) qRT-PCR approach as described elsewhere [30]. Each assay point was repeated four times. The level of U6 snRNAs were served as internal cvontrol. The relative quantification on miR491-5p expression was normalized by using U6 as a reference. Real time qRT-PCR was carried out with iQ5 real-time PCR detection system (Bio-Rad Laboratories, Hercules, CA, USA ) at the following conditions: 42 °C for 5 min and 95 °C for 10 s; 95 °C for 5 s and 60 °C for 10 s and repeated for 40 cycles. The dissociation of the reaction products was conducted from 55 °C to 95 °C as the temperature rose at 0.2 °C per ten seconds.

**Table 1:** Primers used in RT-PCR analysis.

Gene name	GenBank ID	forward primer(5'→3')	reverse primer(5'→3')	Size of product (bp)
*β-acti* *n* ^+^	bc009275	cacactgtgcccatctacga	ctgcttgctgatccacatct	600
*Bcl-X L* ^+^	z23115.1	ggtggttgactttctctcct	gcatctccttgtctacgctt	454
*TP53* ^+^	NM_000546.4	atcacactggaagactccag	ctgacgcacacctattgcaa	741
*β-actin-R* ^++^	bc009275	tccatcatgaagtgtgacgt	ctcaggaggagcaatgatct	161
*Bcl-XL-R* ^++^	z23115.1	ggtggttgactttctctcct	ggcctcagtcctgttctctt	87
*TP53-R* ^++^	NM_000546.4	atcacactggaagactccag	agattctcttcctctgtgcg	103

^+^ primers for standard RT-PCR; ^++^ primers for quantitative RT-PCR.

### 3.4. Transient Transfection

Cells were cultured in 35-mm dishes in Dulbecco’s Modified Eagle Medium (HyClone, South Logan, UT, USA) supplemented with 10% fetal calf serum and incubated in a 37 °C incubator containing 5% CO_2_. Cells were transiently transfected with the indicated plasmid DNAs or synthesized miRNAs using Vigofect reagent (Vigorous Biotechnology, Beijing, China) according to the manual instruction. Briefly, transfected DNAs were first added into a 75 μL of 0.9% NaCl solution. The vigofect reagent (about 1 μL vigofect for 1 μg transfected DNAs or synthesized miRNAs) was diluted into another 75 μL of 0.9% NaCl solution. Then the diluted vigofect mixture was added drop by drop into the diluted DNA solution accompanying with gentle vortexing. After 15 min incubation, the reaction mixture was evenly distributed into the cell culture medium and incubated for 48 h before harvesting.

### 3.5. Western Blot Analysis

The transfected cells were lysed with a lysis buffer containing 1% NP-40, 50 mM Tris-HCl (pH 7.5), 120 mM NaCl, 200 μM NaVO_4_, 1 μg/mL leupeptin, 1 μg/mL aprotinin, and 1 μM PMSF. About 15 μg of cell lysate for each sample was resolved onto 12% SDS-PAGE. After separation, the separated proteins were transferred onto Hybond nitrocellular membrane (Pharmacia, St. Louis, MO, USA). The transferred membrane was first probed with a primary antibody. Then, a secondary antibody labeled with horseradish peroxidase was added to the reaction and finally visualized with an ECL kit (Santa Cruz Biotechnology). 

### 3.6. Apoptotic Assay by Flow Cytometric Analysis

Cell apoptosis was detected by Annexin V-FITC apoptosis detection kit (RuiBang XingYe Sciences & Technology, Beijing, China). After 48-h transfection, the cells were released from the culture plates and washed with 1×PBS. The cell pellet was resuspended with 50 μL binding buffer. Then, about 200 ng Annexin V-FITC was added to the cell suspension and incubated at room temperature for 15 min. About 200 ng Propidium Iodide (PI) was added to the reaction mixture before flow cytometric analysis by FACSARIA flow cytometer (Becton Dickenson, San Jose, CA, USA).

### 3.7. Cell Proliferation assay by Cell-Counting Kit-8 (CCK-8)

Cell proliferation was monitored by CCK-8 as described elsewhere [31]. Briefly, SW1990 cells plated at the density of 5 × 10^4^ cells/mL were grown in a 12 well plate. After 48-h transfection, cell-counting kit-8 solution was added into each well. The viable cells were counted by measuring the amount of color change of WST-8 by spectrophotometer at 450 nm. 

### 3.8. Statistical Analysis

All values were calculated as mean ± standard deviation (SD) from four to six independent experiments. The difference between the assayed group and the standard group was subject to student’s t test. The calculated difference was considered significant as the *p* value < 0.05 or < 0.01.

## 4. Conclusions

Current study revealed a significant down-regulation of miR491-5p in the total RNAs of pancreatic cancer cells *versus* normal pancreatic total RNAs. The down regulation of miR491-5p in pancreatic cancer cells may be a contributive factor for pancreatic cancer development. Nakono *et al.* [21] observed that the miR491-5p could recognize the 3'UTR of Bcl-XL and induced cell apoptosis in human colorectal cancer cells. Current study revealed for the first time that, in addition to Bcl-XL gene, the 3' UTR of tumor suppressor gene TP53 also contains a real targeted site for miR491-5p. miR491-5p may induce cell apoptosis by activating the intrinsic mitochondria mediated pathway in TP53 independent manner. Moreover, our study revealed an important link between miR491-5p and cellular signaling pathways such as STAT3 and PI3K. Our data indicates that a mutual interaction might exist between miR491-5p targeted genes and STAT3 or Akt since down-regulation of Bcl-XL and TP53 gene expressions by miR491-5p inhibits the activities of both STAT3 and Akt in pancreatic cancer SW1990 cells. However, how these signaling molecules being finely regulated and controlled is needed to be further investigated.
